# Preventing Respiratory Syncytial Virus in Infants: A Comparative Review of Nirsevimab and Maternal Respiratory Syncytial Virus Prefusion F (RSVpreF) Vaccination

**DOI:** 10.7759/cureus.94483

**Published:** 2025-10-13

**Authors:** Abdulla J AlZeera, Mohammed A Qamber, Jamal A AlZeera

**Affiliations:** 1 Pediatric Medicine, Dr. Jamal AlZeera Medical Center, Isa Town, BHR; 2 General Medicine, Royal College of Surgeons in Ireland, Busaiteen, BHR; 3 General Medicine, Dr. Jamal AlZeera Medical Center, Isa Town, BHR; 4 Family Medicine, Dr. Jamal AlZeera Medical Center, Isa Town, BHR

**Keywords:** global child health, infant infection prevention, maternal, monoclonal antibody prophylaxis, nirsevimab, pediatric infectious diseases, respiratory syncytial virus (rsv), rsvpref vaccine (abrysvo), rsv vaccination, vaccine implementation

## Abstract

Respiratory syncytial virus (RSV) is a major cause of lower respiratory tract disease in early infancy. Preventive options have recently expanded beyond selective palivizumab use to two population-level strategies: infant administration of the long-acting monoclonal antibody nirsevimab and maternal immunization with an RSV prefusion F (RSVpreF) vaccine during late pregnancy. This review synthesizes current evidence on the efficacy, safety, durability, and programmatic considerations of both approaches, with attention to comparative strengths and real-world implementation. We outline clinical trial findings, early post-licensure effectiveness and safety data, and health-economic considerations relevant to diverse health systems. We also discuss limitations of the evidence base and propose priorities for surveillance and comparative research to guide policy decisions. The goal is to provide clinicians and policymakers with a concise, practice-oriented appraisal of how these strategies can be integrated to reduce RSV burden in the first months of life.

## Introduction and background

Respiratory syncytial virus (RSV) is the leading cause of acute lower respiratory tract infection (LRTI) in infants and young children, accounting for an estimated 3.6 million hospitalizations and approximately 100,000 deaths globally each year [1]. Nearly all children are infected with RSV by two years of age, and the highest burden of severe disease occurs in infants under six months, particularly in low- and middle-income countries (LMICs) where access to advanced supportive care is limited [2]. Despite the magnitude of this burden, preventive options remained limited for decades, with palivizumab, the only licensed monoclonal antibody for RSV prophylaxis, restricted to high-risk infants due to cost and the need for monthly administration [3].

The development of two novel immunoprophylactic strategies has transformed the prevention landscape: nirsevimab, a long-acting monoclonal antibody administered directly to infants, and the maternal respiratory syncytial virus prefusion F (RSVpreF) vaccine (Abrysvo, Pfizer Inc., New York, NY), which induces maternal antibodies that cross the placenta and protect newborns. Both have shown significant efficacy in randomized controlled trials, offering, for the first time, the possibility of broad protection against RSV in otherwise healthy infants [4-6].

Nirsevimab is engineered with an Fc modification that prolongs its half-life, enabling a single intramuscular dose to provide protection for at least five months, covering an entire RSV season [7,8]. The HARMONIE trial, the largest to date, demonstrated substantial reductions in RSV-related hospitalizations in term and late preterm infants [5]. In parallel, maternal immunization with the RSVpreF vaccine administered during late pregnancy reduced medically attended severe RSV illness in infants under six months of age, with added maternal protection during pregnancy [6].

Both strategies received regulatory and programmatic endorsement beginning in 2023 in the United States and Europe, with rapid global policy evolution since then [8-11]. As adoption expands, key questions include comparative effectiveness across populations, optimal implementation strategies, cost-effectiveness under diverse epidemiology and pricing, and the balance of benefits and risks in special populations. Programmatic challenges, antenatal vaccine uptake, monoclonal antibody pricing and supply, and delivery logistics will strongly influence real-world impact [9-14].

We synthesize current data on nirsevimab and maternal RSV vaccination, examine comparative strengths and limitations, and highlight knowledge gaps to inform clinical and policy decision-making, taking a clinical and programmatic perspective for pediatric, obstetric, and public-health audiences with global applicability and practical guidance for both high- and low-resource settings.

Scope and methods (narrative review)

We conducted a targeted narrative review of peer-reviewed and official sources in PubMed/MEDLINE and Embase and via WHO/CDC/FDA websites (January 2020-September 2025), using combinations of “RSV”, “nirsevimab”, “maternal RSV vaccine”, “RSVpreF”, “infant”, “hospitalization”, “effectiveness”, and “cost-effectiveness”. We prioritized randomized trials, large pragmatic/observational evaluations on infants ≤6 months, and maternal vaccination during late pregnancy.

## Review

Efficacy of nirsevimab in infants

Clinical Trial Data

Nirsevimab has demonstrated robust efficacy in preventing RSV-associated lower respiratory tract disease (LRTD) and hospitalization in both term and preterm infants. The MELODY trial (phase 3 RCT) reported vaccine efficacy of 74.5% (95% CI, 49.6-87.1) against medically attended RSV LRTD through 150 days, and 62.1% (95% CI, -8.6 to 86.8; P = 0.07) against RSV-related hospitalization [5]. The pragmatic HARMONIE trial found 83.2% efficacy (95% CI, 67.8-92.0; P < 0.001) against RSV-related hospitalization and 75.7% (95% CI, 32.8-92.9; P = 0.004) against very severe RSV LRTD [9]. Importantly, these protective effects were observed in otherwise healthy late preterm and term infants, a population previously without access to prophylaxis. These key outcomes are summarized in Table 1.

**Table 1:** Key Clinical Trial Outcomes of Nirsevimab and Maternal RSVpreF Vaccine in Preventing RSV Disease in Infants Data extracted from phase 3 trials MELODY, HARMONIE, and MATISSE [5,8,9]. GA = gestational age; LRTD = lower respiratory tract disease.

Intervention	Trial (Year)	Population	Efficacy Against RSV LRTD	Efficacy Against RSV Hospitalization	Duration of Protection	Safety Profile
Nirsevimab	MELODY (2022) [5]	Healthy term and preterm infants (≥29 weeks GA)	74.5% ↓ medically attended RSV LRTD	62.1% ↓ hospitalizations	~5 months	Adverse events similar to placebo
Nirsevimab	HARMONIE (2023) [9]	>8,000 term and late preterm infants	77% ↓ very severe RSV LRTD	83% ↓ RSV hospitalizations	~5 months	No significant safety concerns
Maternal RSVpreF Vaccine (Abrysvo)	MATISSE (2023) [8]	Pregnant women (24-36 weeks GA), infant follow-up	81.8% ↓ severe RSV LRTD (≤90 days); 69.4% ↓ (≤180 days)	69.4% ↓ severe RSV LRTD (≤180 days)	Up to 6 months	Local/systemic reactions; no ↑ obstetric/neonatal complications

Across MELODY and the pragmatic HARMONIE evaluation, point estimates were consistent despite differences in enrollment strategy, setting, and primary endpoints, supporting external validity for routine-care programs. In MELOODY, efficacy was robust across RSV A and B, and severity markers (oxygen requirement, escalation of care) trended lower among breakthrough cases, aligning with hospitalization reductions observed in HARMONIE [4,5,9]. Early real-world program evaluations likewise report large reductions in RSV hospitalizations consistent with trial efficacy [14,15]. Extension and pooled analyses further support a favorable benefit-risk balance, including in infants near the lower gestational-age boundary, with pharmacokinetic bridging informing extrapolation to certain high-risk subgroups [16]. Collectively, these data justify season-wide protection from a single infant dose when timed to local RSV circulation [4,7,16].

Real-World Effectiveness

Post-licensure evaluations in Europe and the United States have corroborated trial results. Surveillance data suggest effectiveness ranging from 70-80% against RSV-related hospitalization, with up to 98% protection against severe RSV disease requiring intensive care [7,14]. These findings highlight the consistency of benefit across both controlled and real-world settings, supporting nirsevimab’s potential role in universal seasonal prophylaxis. Early observational cohorts also report reductions in emergency department visits for bronchiolitis, shorter lengths of stay among breakthrough cases, and sustained effectiveness across early and late peaks within the same season, findings that mirror pragmatic-trial experience [15]. In addition, pooled analyses of randomized trials corroborate both efficacy and a favorable safety profile [16], and early pharmacovigilance assessments remain reassuring as uptake expands [17]. Early U.S. and European program data mirror trial performance. U.S. surveillance reported ≈90% effectiveness against RSV-associated hospitalization during the first season of use, with a median of 45 days from dose to symptom onset in cases, consistent with expected antibody decay over time [14]. Population-based evaluation from Galicia, Spain, showed substantial reductions in RSV hospitalizations and severe disease during universal roll-out, reinforcing impact at scale [15]. These reports also describe operational lessons (birth-hospital administration, rapid eligibility verification, and linkage with immunization registries) that can be adapted to differing health-system architectures [14,15].

Pharmacology and Dosing Considerations

Nirsevimab’s extended-half-life Fc engineering underpins season-long neutralizing activity from a single intramuscular dose. In practice, programs schedule administration at the birth hospitalization or the earliest outpatient well-baby visit preceding the expected RSV season. Weight-based dosing and alignment to local season onset are central operational levers; these choices aim to minimize missed opportunities while preserving consistent exposure through the highest-risk months [5,9], consistent with product labeling, pharmacokinetic, and dosing information [18]. The extended half-life of nirsevimab (engineered Fc changes) sustains neutralizing activity through a single season. Label guidance supports weight-banded dosing administered at birth, hospitalization, or the earliest well-baby visit before season onset, with alignment to local epidemiology to minimize missed opportunities [18]. Programs commonly combine standing orders, EHR prompts, and discharge checklists to capture eligible newborns while preserving coverage for out-of-season births via early outpatient visits [5,7,18].

Maternal vaccination and infant outcomes

Efficacy in Clinical Trials

Maternal immunization with the RSVpreF vaccine has also demonstrated substantial efficacy. The MATISSE trial reported vaccine efficacy of 81.8% against medically attended severe RSV LRTD within 90 days after birth (99.5% CI, 40.6-96.3) and 69.4% within 180 days (97.58% CI, 44.3-84.1) [8]. These benefits extend to the mother, who receives protection from RSV illness during pregnancy, a period of heightened vulnerability. Antibody transfer kinetics support the highest infant titers following vaccination in the late second to third trimester, consistent with the gestational window used in the pivotal trial [8]. The magnitude of protection in MATISSE for severe infant RSV LRTD within 90 and 180 days dovetails with biological expectations for transplacentally transferred IgG and measured infant titers following third-trimester vaccination [5]. In parallel, maternal protection during pregnancy reduces the risk of RSV illness at a time of heightened respiratory vulnerability, an additional programmatic benefit not conferred by infant monoclonal strategies [5,9-11].

Implementation Considerations

Despite these benefits, vaccine uptake is influenced by antenatal care access and timing. Optimal protection requires administration between 24 and 36 weeks of gestation (per trial parameters), with program guidance commonly recommending 32-36 weeks to balance maternal safety considerations with efficient transplacental transfer [9,10]. Missed antenatal windows, late presentation to care, and cancellations can reduce infant coverage. Additionally, cultural and health-system factors affecting maternal vaccine acceptance can significantly influence programmatic success [9,10]. As with nirsevimab, the trial results and safety outcomes of maternal vaccination are outlined in Table 1. Because effective transfer depends on timing, guidance emphasizes vaccination in late pregnancy (often 32-36 weeks) to balance maternal safety and infant antibody levels [9-11]. Missed antenatal windows and late presentation to care can meaningfully erode infant coverage; programs can mitigate this via point-of-care scheduling at glucose screening/third-trimester visits, reminder/recall systems, and culturally tailored counseling to address vaccine hesitancy [9-11]. Perinatal outcomes analyses in routine care remain reassuring when maternal vaccination is administered within the recommended third-trimester window [19]. Early national coverage reports highlight differential uptake between infant nirsevimab and maternal vaccination, underscoring the importance of both platforms to close protection gaps [20].

Transplacental Transfer and Coverage Gaps

Placental transfer may be attenuated with earlier preterm delivery, potentially shortening the infant’s window of passive protection; for settings with higher preterm-birth prevalence, adjunct infant strategies (e.g., nirsevimab at discharge) can complement maternal immunization by providing direct postnatal protection. Conversely, where antenatal attendance and on-time vaccination are high, maternal vaccination efficiently front-loads protection during the period of peak vulnerability [8-10]. For infants delivered preterm, reduced transfer duration can shorten the window of passive protection; a pragmatic approach is maternal vaccination when eligible plus postnatal nirsevimab at discharge for those with insufficient transplacental antibody, aligning with policy recommendations [9-11,18].

Comparative effectiveness

**Figure 1:**
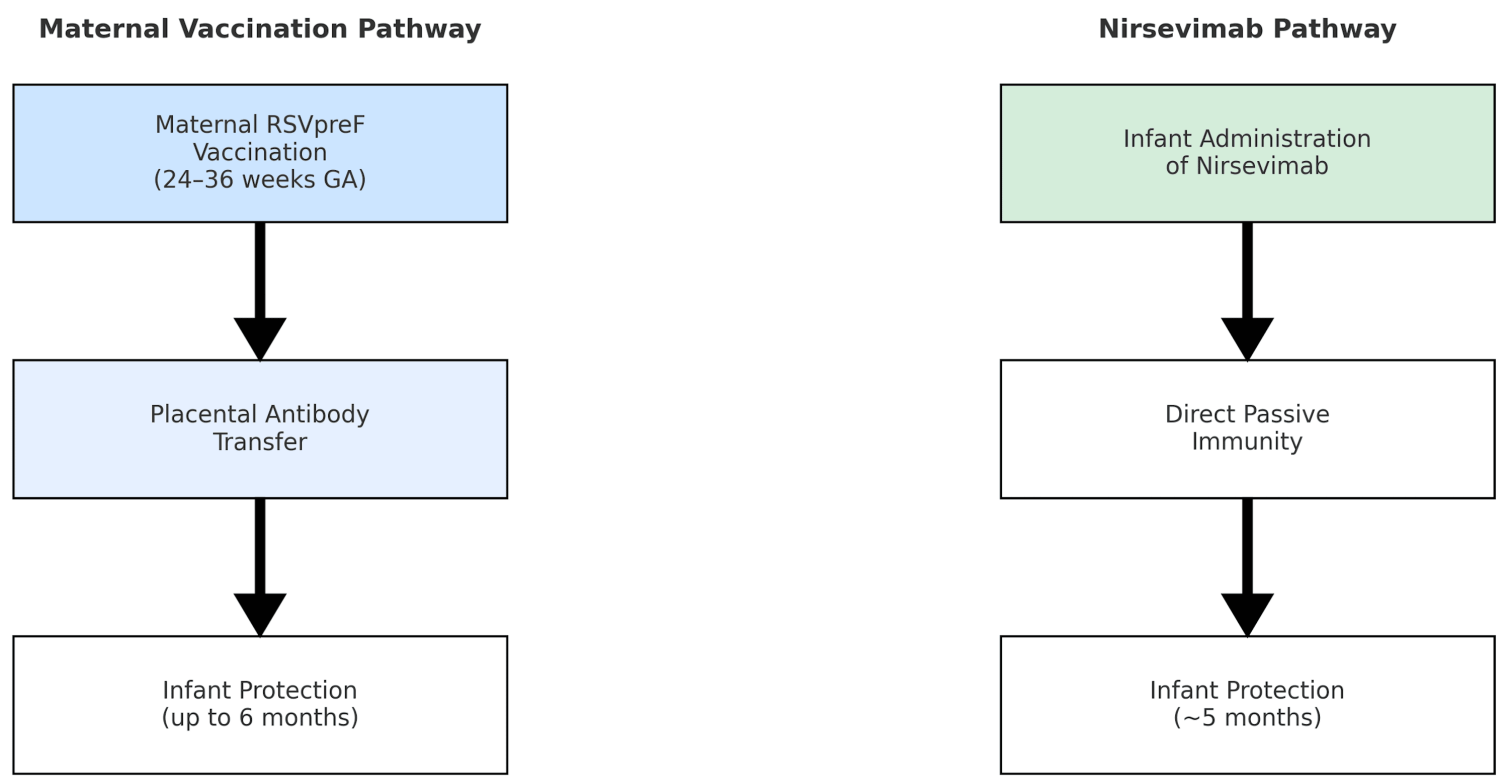
Pathways of RSV Prevention in Early Infancy Through Maternal Vaccination and Nirsevimab Maternal RSVpreF vaccination administered during 24-36 weeks of gestation induces maternal antibody production with transplacental transfer to the infant, conferring protection for up to six months. Nirsevimab, a long-acting monoclonal antibody administered directly to the infant, provides protection for approximately five months (one RSV season). GA = gestational age; RSV = respiratory syncytial virus.

Direct comparisons between nirsevimab and maternal vaccination are limited, as trials differed in population characteristics, timing of administration, and study endpoints. Nirsevimab offers direct infant protection irrespective of maternal factors, while maternal vaccination confers dual maternal-infant benefits but depends on antenatal uptake and correct timing. Modeling studies suggest that combined strategies may offer complementary advantages, particularly in high-burden regions [13], and international policy/value frameworks emphasize context-specific adoption within maternal-child health platforms [11,12,18]. Head-to-head trials are absent, but triangulation across RCTs, pragmatic data, value frameworks, and policy statements suggests complementary strengths: nirsevimab provides direct, reliable infant protection independent of antenatal attendance, whereas maternal vaccination provides dual mother-infant benefit when late-pregnancy care is accessible and timely [11,12,18]. Health-system choice should therefore weigh antenatal coverage, birth-facility readiness, seasonality, and pricing; mixed strategies can be justified in high-burden or low-coverage settings to maximize protected infant-months [11,12]. Figure 1 illustrates these distinct prevention pathways, highlighting differences in timing, mechanism, and programmatic considerations.

Safety and pharmacovigilance

Both interventions have demonstrated favorable safety profiles. In the HARMONIE trial, adverse events related to nirsevimab were uncommon and comparable to placebo [9]. Maternal RSV vaccination was associated with injection-site reactions and transient systemic symptoms but no significant increase in obstetric or neonatal complications [8]. Nonetheless, continued pharmacovigilance is warranted to detect rare events as uptake expands. Early reports from post-marketing surveillance have not identified concerning safety signals [10,12]. Programs should leverage existing safety-signal platforms and pre-specified adverse event of special interest (AESI) definitions, link coverage databases to outcomes, and publish periodic safety summaries to sustain confidence in both strategies [9,10,12], with emerging perinatal outcomes analyses in routine use remaining reassuring [19]. Across pooled infant trial datasets, adverse events and AESI rates remained comparable to placebo, and sequence monitoring has not identified concerning resistance patterns affecting the prefusion-F epitope [16]. For maternal immunization, pharmacovigilance summaries and perinatal outcomes analyses in routine care have not shown increased risk of obstetric complications (including preterm birth) when administered in the recommended gestational window, though ongoing monitoring remains essential [17,19]. 

Cost-effectiveness and implementation challenges

Economic analyses indicate that both strategies may be cost-effective, but outcomes vary by region and depend on RSV burden, intervention pricing, and healthcare utilization patterns. Nirsevimab’s relatively high production cost is a barrier in low-resource settings, while maternal vaccination faces challenges of antenatal coverage and acceptance. Integrated approaches, including tiered pricing mechanisms, advance procurement, and alignment with existing immunization schedules, may be necessary to ensure equitable access [13,14]. Table 1 and Figure 1 together emphasize that optimal strategy selection will ultimately depend on health-system resources, antenatal service reach, local seasonality, and implementation feasibility. In practice, birthing-hospital administration of nirsevimab can mitigate missed opportunities where prenatal attendance is inconsistent, while strong antenatal platforms can efficiently deliver maternal vaccination with dual maternal-infant benefit [9,10,13,14,20].

Economic work emphasizes that results are context-dependent: baseline RSV burden, product pricing, payer mix, and care-seeking patterns drive ICERs and budget impact. Value-framework analyses and national guidance converge on the need for tiered pricing and pooled/advanced procurement to enable equitable access, while program design (birth-hospital vs antenatal delivery) determines marginal coverage gains at scale [11,12,18]. Early coverage reports reveal heterogeneity by state and population group, pointing to operational and equity opportunities for both platforms [20]. 

Limitations, recommendations, and future directions

Limitations of the Evidence

Absence of head-to-head trials limits comparative inference. Effectiveness estimates in observational studies are sensitive to residual confounding and testing intensity; subgroup data in infants with complex comorbidities and second-season prevention strategies remain comparatively sparse. Real-world performance may vary with RSV seasonality and circulating genotypes, reinforcing the need for routine effectiveness monitoring [7-10,12]. Accumulating RWE, pooled analyses, and policy documents help close gaps, but priorities remain: comparative-effectiveness evaluations (including mixed strategies), multi-season durability and re-dosing studies, and equity-focused implementation research in low-resource settings [14-18,20,21]. To aid implementation, we summarize the principal evidence gaps, why they matter for practice, immediate actions for programs, and priority research needs in Table 2.

**Table 2:** Key Evidence Gaps and Actionable Next Steps for Infant RSV Prevention Programs Evidence and implications summarized from MELODY, HARMONIE, MATISSE, early pharmacovigilance and policy/economic sources [5,6,8-10,12-17]. AESI = adverse events of special interest; CHD = congenital heart disease; CLD = chronic lung disease; LMICs = low- and middle-income countries; PK/PD = pharmacokinetic/pharmacodynamic.

Evidence Gap / Limitation	Why It Matters	Recommended Actions (Practice/Policy)	Priority Research Directions	
No head-to-head comparison of nirsevimab vs maternal RSVpreF	Uncertain relative benefit by context and population; complicates policy selection	Use context-specific criteria (antenatal coverage, birth-facility readiness, pricing) to choose or combine strategies	Pragmatic cluster-randomized or stepped-wedge trials; linked registry comparative-effectiveness studies [5,6,8,9,13]	
Limited data in high-risk subgroups (extreme prematurity, CLD, CHD, immunocompromise)	Effect size and dosing/timing may differ; highest-risk infants stand to benefit most	Default to strategies that ensure coverage at birth/early infancy; specialist pathways for complex infants	Subgroup registries; adaptive platform trials; pooled IPD meta-analyses [5,6,9,12]	
Durability across variable seasonality; second-season protection	Program timing and re-dosing decisions hinge on local epidemiology	Align dosing with local season onset; develop clear second-season criteria	Multi-season cohorts; PK/PD modeling; effectiveness by season timing [5,6,12,15]	
Delivery platform constraints (antenatal uptake vs birth-hospital coverage)	Coverage gaps if timing/access are poor for one platform or the other	Standing orders at birth hospitals; third-trimester scheduling prompts; missed-opportunity audits	Implementation trials of workflow bundles; human-factors studies [9,10,13,14]	
Pricing and supply predictability (both products)	Equity and scale-up feasibility depend on sustainable procurement	Tiered pricing; pooled/advance procurement; seasonal stock planning	Budget-impact analyses; full-value assessments in LMICs [13,14,17]	
Rare safety events detection and communication	Public confidence and sustained uptake require transparent safety monitoring	Define AESI; link coverage to outcomes; regular public safety updates	Enhanced pharmacovigilance (VSD-like); signal validation networks [8,10,12]	
Viral evolution and potential antigenic drift	Could attenuate neutralization and real-world effectiveness	Genomic surveillance; periodic laboratory neutralization panels	Escape-risk mapping; correlates of protection studies [16]	
Maternal vaccine acceptance and counseling quality	Determines real-world coverage and equity of protection	Provider training; culturally tailored counseling; reminder/recall systems	Behavioral trials; communication science research [10,14,17]	

Recommendations for Practice and Policy

Select strategy based on antenatal coverage and delivery platforms: where prenatal uptake and on-time third-trimester visits are high, maternal vaccination is efficient and provides maternal protection; where antenatal access is limited or late, infant nirsevimab at birth, hospitalization, or early well-visit provides predictable coverage [9,10,13,14]. In mixed-coverage settings, a hybrid approach (maternal vaccination as default plus nirsevimab for infants with missed antenatal windows, out-of-season births, or early preterm delivery) can maximize protected infant-months without duplicating efforts [9-12]. Programs should institutionalize missed-opportunity minimization via standing orders in newborn units, discharge checklists, EHR prompts, and real-time eligibility flags in immunization registries; antenatal services should use point-of-care scheduling (32-36 weeks), reminder/recall systems, and culturally tailored counseling to address hesitancy and language barriers [9-12,14,20]. Pharmacy and supply teams should align seasonal stock to local RSV epidemiology, establish buffer inventory for surges, and define fallback outpatient pathways for infants discharged outside the dosing window; weight-banded dosing and label guidance should be embedded in order sets to reduce errors and streamline throughput [6,9-12,18]. Equity requires proactive outreach for rural/remote births, linkage with community health workers and home-visiting programs, and removal of point-of-care cost barriers where applicable; dashboards should track coverage by gestational age, birth setting, neighborhood deprivation, and ethnicity to identify gaps and drive targeted QI cycles [11-14,20]. Finally, communication materials should integrate safety messaging grounded in trial and post-authorization data to sustain confidence while directing clinicians to adverse-event reporting channels and periodic public summaries [5,7,16,17,19].

Operational metrics to monitor (not exhaustive): dose-before-discharge rate in eligible newborns; maternal vaccine uptake at 32-36 weeks; time-to-dose from birth; missed-opportunity rate; coverage by high-risk subgroup (e.g., late preterm); serious-AE reporting completeness; and RSV-associated hospitalization rate among age <6 months across seasons [9-12,14-17,20].

Future Directions

Priorities include pragmatic comparative-effectiveness evaluations of strategy choice and sequencing (e.g., maternal vaccine vs nirsevimab vs targeted combination) using cluster or stepped-wedge designs embedded in routine care; multi-season durability studies to define protection across atypical peaks and criteria for second-season prophylaxis in high-risk children; and subgroup analyses (extreme prematurity, CHD/CLD, immunocompromise, growth-restricted neonates) with pooled individual-patient data to refine dosing and timing [11,12,14-16,18]. Expanded pharmacovigilance should link immunization registries, birth records, and outcomes to detect rare events with timely public reporting; perinatal outcome surveillance for maternal vaccination should continue as coverage scales, with clear risk communication when signals arise [17,19]. Methodological work should map correlates of protection (maternal and infant neutralizing titers vs outcomes), evaluate impacts on downstream utilization (ED visits, ICU days, antibiotic use), and integrate genomic surveillance to monitor antigenic drift relevant to prefusion-F targeting [16-18]. Health-economic studies should incorporate local prices, payer mix, and delivery costs, with budget-impact scenarios for tiered pricing and pooled/advanced procurement to inform sustainable national adoption; coverage analytics (by geography and population) should feed back into iterative program design [11,12,18,20]. Finally, international guidance and policy communications should continue to emphasize context-specific adoption within maternal-child health platforms, including pathways for LMIC implementation and equity safeguards as supply expands [11,21].

## Conclusions

Nirsevimab and maternal RSVpreF vaccination have transformed infant RSV prevention by offering safe, effective strategies that extend protection beyond high-risk groups. Nirsevimab provides reliable, season-long direct infant protection with a single dose, while maternal vaccination confers dual maternal-infant benefits when administered during late pregnancy. Choice of strategy should reflect local antenatal coverage, delivery platforms, and resource constraints.

Evidence gaps, particularly the lack of head-to-head comparisons, limited subgroup data, and evolving real-world effectiveness, justify ongoing surveillance and pragmatic comparative studies. Health systems should consider context-specific implementation, potential complementarity of approaches, and equity-minded financing to maximize impact on early-life RSV morbidity.
